# ARACRA: Automated RNA-seq Analysis for Chemical Risk Assessment

**DOI:** 10.34133/csbj.0186

**Published:** 2026-08-19

**Authors:** Shubh Sharma, Saurav Kumar, Judit Biosca-Brull, Deepika Deepika, Vikas Kumar

**Affiliations:** ^1^ Institut de Recerca Biomèdica Catalunya Sud (IRBCatSud), Department of Chemical Engineering, Universitat Rovira I Virgili, Tarragona, Spain.; ^2^ German Federal Institute for Risk Assessment (BfR), Berlin, Germany.; ^3^ Universitat Rovira i Virgili, Pere Virgili Research Institute, Center of Environmental, Food and Toxicological Technology (TECNATOX), Reus, Spain.; ^4^ Universitat Rovira i Virgili, Department of Biochemistry and Biotechnology, Tarragona, Spain.

## Abstract

Transcriptomics data captures genome-wide gene expression changes through *in vitro* or *in vivo* studies and can be used in chemical risk assessment for characterizing and identifying the effects of chemicals. While several bioinformatics tools and pipelines address discrete steps of the RNA sequencing (RNA-seq) workflow, a complete end-to-end framework from raw FASTQ files to transcriptomic point of departure in a streamlined way is unavailable and limits its accessibility to researchers without computational expertise. To address this gap, we present ARACRA, a fully automated RNA-seq analysis pipeline including entire transcriptomics workflow from raw FASTQ files to the transcriptomic point of departure with human-in-the-loop review process. Overall, the analysis is performed in 2 phases: Phase 1 carries out the acquisition of raw reads, prealignment quality control, alignment to reference genome, and quantification of gene expression, whereas Phase 2 performs statistical analysis including differential gene expression analysis and dose–response modeling. ARACRA was validated against a publicly available dataset (GSE271332) comprising 286 samples from MCF-7 cells exposed to bisphenol A (BPA) and 11 data-poor alternatives. The potency ranking of the chemicals were consistent with original results in which 2,4’BPA and BPA were found to be most transcriptionally active chemicals. Overall, ARACRA facilitates end-to-end analysis of RNA-seq data through an interactive web-based application developed on Nextflow (workflow management system) and Streamlit (an open-source python framework for graphical user interface) that minimizes computational complexities and ensures correct downstream processing.

## Introduction

Transcriptomics, a comprehensive study of total RNA (coding and noncoding) within a cell or tissue, has become a powerful approach to characterize gene regulation and associated changes to external stressors and conditions [1,2]. Several strategies are available for measuring transcriptome changes, including microarrays, whole RNA sequencing (RNA-seq), and targeted RNA-seq, which offer higher sensitivity and resolution [3]. In toxicology and risk assessment, RNA-seq in particular has emerged as a new approach methodology (NAM) for biomarker discovery, capturing molecular signals that can be associated with perturbed biological pathways relevant to adverse health outcomes [4]. Analyzing the changes in gene expression upon exposure can support multiple applications: (a) Identification of shared transcriptional signatures and pathway perturbation patterns can support chemical grouping and read-across, (b) constructing dose–response curves from expression profiles at different exposure concentrations for deriving Point of Departure (PoD), and (c) benchmark dose (BMD) contributing to quantitative risk assessment [5,6]. Transcriptomics analysis, as a NAM, is being advanced under European Union chemical- and material-safety initiatives, alongside quantitative structural-activity relationship-based hazard prediction [7], physiologically based kinetic modeling [8], and project-level programs establishing harmonized NAM and testing frameworks for emerging chemicals and advanced materials [9,10].

Numerous bioinformatics platforms and computational pipelines have been developed to facilitate the processing and analysis of HTTr (high-throughput transcriptomics) data. Examples include ZARP (Zavolan-Lab Automated RNA-Seq Pipeline) and metaTP; both integrate different bioinformatics tools for RNA-seq data analysis [11,12]. ZARP is used for generating gene expression files, while meta-TP analyze the data from Sequence Read Archive (SRA) to find differentially expressed genes (DEGs). For upstream read processing, nf-core/rnaseq provides a community-curated Nextflow pipeline covering quality control (QC), alignment, and gene quantification with comprehensive Quality Control reporting but does not perform differential expression or dose–response modeling [13]. Galaxy offers an accessible web-based platform for general-purpose RNA-seq analysis but requires multiple manual wrapping of tools and currently does not incorporate dose–response modeling [14]. For dose–response modeling specifically, BMDExpress 2 [15], maintained by the National Toxicology Program, supports BMD analysis across multiple microarray and sequencing platforms with Gene Ontology (GO) and Reactome pathway classification. BMDx [16] and its successor BMDx2 [17] provide R-Shiny interfaces for BMD computation with adverse outcome pathway (AOP)-based mechanistic annotation. DRomics [18] offers a specialized R framework for omics-scale dose–response analysis with built-in model selection and BMD calculation. These tools collectively cover the full analytical steps from raw reads to biological interpretation. However, in practice, a complete toxicogenomic dose–response analysis from FASTQ files to transcriptomic points of departure (tPODs) requires sequential use of multiple independent tools. Each transition between tools introduces manual format conversions, parameter respecification, and potential for inconsistency. Furthermore, the growing use of targeted TempO-Seq in high-throughput toxicogenomics introduces additional analytical requirements like probe-based alignment, multiprobe gene aggregation, and platform-specific QC that are not natively supported by general-purpose RNA-seq pipelines. TempO-Seq quantifies gene expression by sequencing ligated detector oligonucleotide pairs hybridized to predefined gene-specific regions directly from crude lysates. As the assay generates reads corresponding only to known probe sequences, data processing is simplified to direct mapping and counting without conventional alignment or transcript assembly steps [19].

In this line, Verheijen *et al*. [20] developed the Regulatory Omics Data Analysis Framework (R-ODAF), a framework specifically designed for omics data analysis in regulatory applications. R-ODAF provides an automated pipeline for regulatory-grade transcriptomic analysis including differential expression and robust gene filtering. The Health Canada version of R-ODAF provides an automated, regulatory-grade pipeline from raw FASTQ files through alignment and differential expression analysis, producing outputs formatted for downstream BMD modeling; however, dose–response analysis and tPoD derivation require separate tools and are not performed within the framework itself. DoseRider [21] provides a web application and R package for linear and nonlinear dose–response modeling that estimates BMDs directly at the level of biological pathways and gene-set signatures using generalized mixed-effect models, additionally introducing trend-change doses as a descriptor of complex dose–response behavior.

To address this, we developed ARACRA (Automated RNA-seq Analysis for Chemical Risk Assessment), a Nextflow DSL2 pipeline with an interactive Streamlit web interface that integrates the complete toxicogenomics workflow into a single, reproducible framework. ARACRA does not provide any new algorithm for RNA-seq data analysis, rather, it orchestrates well-established bioinformatics tools to facilitate end-to-end analysis for researchers without programming expertise. ARACRA provides: (a) SRA data retrieval through alignment, QC, and quantification for both RNA-seq and TempO-Seq platforms; (b) interactive multilayer QC with human-in-the-loop outlier review; (c) integrated DRomics dose–response modeling with quality filters; (d) pathway-level tPOD computation across GO, Kyoto Encyclopedia of Genes and Genomes (KEGG), and Molecular Signatures Database (MSigDB) gene-set collections for targeted species (*Homo sapiens*) with multiple tPOD derivation methods; and (e) a web interface enabling interactive parameter tuning and result visualization. For validation, ARACRA was applied to a publicly available TempO-Seq dataset from Beal *et al*. [22] examining bisphenol A (BPA) and 11-alternatives exposure in MCF-7 cells.

## Implementation

### Architecture overview

ARACRA is implemented in Nextflow DSL2 workflow management system (version 25.10.4) with R scripts (version 4.4.3) for statistical analysis and a Python-based Streamlit (version 1.55.0) web interface for user interaction. The pipeline is divided into 2 phases separated by an interactive QC gate. Phase 1 includes read acquisition, prealignment QC, alignment, postalignment QC, gene quantification, and outlier detection. A detailed QC report is generated at the end of Phase 1 for users to visually inspect the quality of data and remove poor quality samples from downstream processing. Phase 2 includes differential expression of gene analysis and dose–response modeling for biological interpretation of the RNA-seq data. This separation ensures interactive data visualization and inspection to ensure that high-quality data are processed, excluding any technical artifacts under the user’s supervision.

**Fig. 1.**
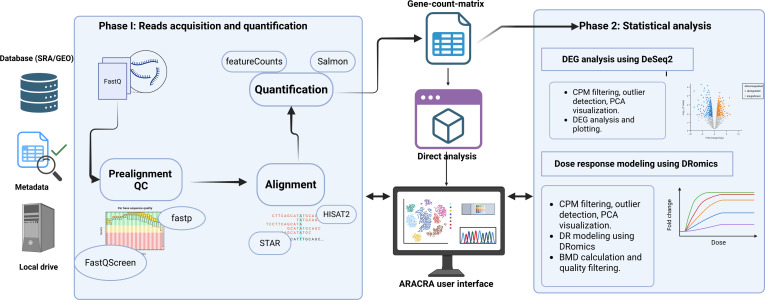
Overall architecture of ARACRA. Two-phase workflow with an interactive quality control (QC) checkpoint: Phase 1 performs read processing, QC, alignment, postalignment QC, and quantification to generate a count matrix and QC reports; Phase 2 conducts differential expression and dose–response analysis. The workflow supports a Direct Analysis mode for precomputed count data with user-guided QC and inspection.

ARACRA additionally supports a Direct Analysis mode in which users can provide a preexisting count matrix and metadata file, bypassing the preprocessing phase entirely. In this mode, a PCA (principle component analysis)-based QC check runs first, followed by the same interactive review gate before analysis proceeds. It also allows the user to analyze TempO-Seq data (targeted RNA-seq data), which uses a specific probe set to identify transcriptomic perturbations following chemical exposure. Figure 1 represents the overall architecture of ARACRA.

### Core workflow

In Phase 1, raw sequencing reads are retrieved from the databases such as Gene Expression Omnibus (GEO) or SRA, using SRA Toolkit or local drive on the computer. It then undergoes prealignment QC steps such as: adapter trimming, assessing the phred score, contamination detection, and removal using fastp [23] and FastQ Screen [24]. A Phred score indicates the measure of base quality in sequencing, i.e., the probablity that the nucleotide was called incorrectly. The reads are then aligned to reference genome using HISAT2 (Hierarchical Indexing for Spliced Alignment of Transcripts 2) or STAR (Spliced Transcripts Alignment to a Reference) [25,26] depending on available computational resources. It also offers an alignment option for TempO-Seq data that, instead of using the entire genome and building a full index, uses a subset of the transcriptome for index construction and alignment. Quantification is performed using tools such as featureCounts and Salmon [27,28] generating gene-level count matrices. For postalignment QC, ARACRA integrates RSeQC [29], Picard, and QualiMap [30], with automated flagging of low-quality samples based on thresholds adapted from R-ODAF pipeline. MultiQC [31] then summarizes the overall quality report in 1 consolidated file available for user to inspect and download. Also, exploratory data analysis is done to detect outliers, in which initial normalization using variance stabilizing transformation, batch correction using Combat-Seq [32], and PCA analysis of overall samples is done with user’s oversight. Batch correction is done only for visualization, while final batch correction is done internally by DESeq2 by introducing batch as a covariate so that it is not overcorrected.

**Fig. 2.**
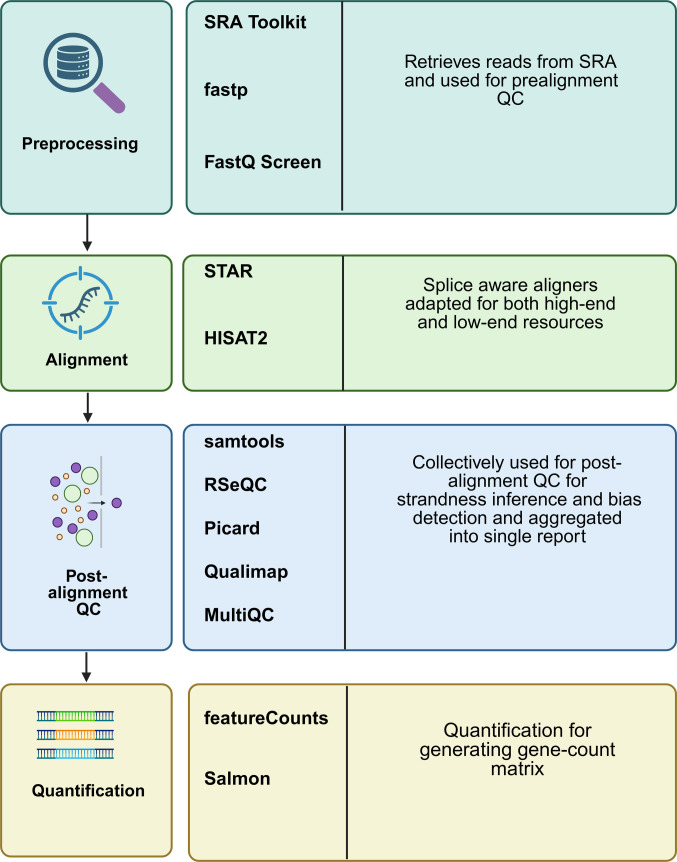
Bioinformatics tools integrated within the ARACRA pipeline and their functions. Summary of tools used across preprocessing, alignment, postalignment QC, and quantification steps, highlighting their roles in read processing, quality assessment, alignment, and gene expression quantification.

Before passing the gene count matrix for statistical analysis in Phase 2, CPM (counts per million)-based filtering removes low-expression noise genes from the matrix. DeSeq2 [33] is used for identifying DEGs both standard treated-*vs.*-control and per-dose comparisons. Dose–response modeling is performed with DRomics [18], fitting 5 candidate models selected by Akaike information criterion (AICc), from which BMD values are derived alongside quality filters for extrapolation, confidence interval (CI) width, and fold-change magnitude. CIs (benchmark dose lower confidence limit and benchmark dose upper confidence limit) are estimated by the bmdboot function of DRomics (1,000 iterations as default; BMDL and BMDU taken as the 2.5th and 97.5th percentiles, i.e., a 95% interval); only BMDs with a defined bootstrap CI are retained. Pathway-level tPODs are computed following the NTP (National Toxicology Program) 2018 [34] framework by mapping quality-filtered BMD genes to GO [35,36], KEGG [37,38], MSigDB Hallmark, and MSigDB C2 pathway collections [39], complemented by distribution-based tPOD estimates from the ranked gene-level BMD distribution [6,40]. Distribution based methods include Rank 25 tPOD, 5th percentile, 10th percentile, the first-mode (maximum-curvature) estimate, and the median gene BMD. Figure 2 summarizes the list of tools integrated in ARACRA along with their purposes. Detailed description of each step can be found in Supplementary Materials File S1.

### Resource management, initial setup, and reproducibility

The setup.sh installation script initiates the setup of ARACRA and subsequent dependencies. It autodetects system resources (central processing unit cores, available random-access memory [RAM], and disk space) and configures the Nextflow process labels accordingly. Lightweight processes such as fastp trimming are parallelized across available cores, while memory-intensive processes such as STAR alignment are limited to 2 concurrent instances to prevent out-of-memory failures. On systems with less than 32-GB RAM, HISAT2 is automatically recommended over STAR, and the generated configuration reflects this recommendation.

### Web Interface

**Fig. 3.**
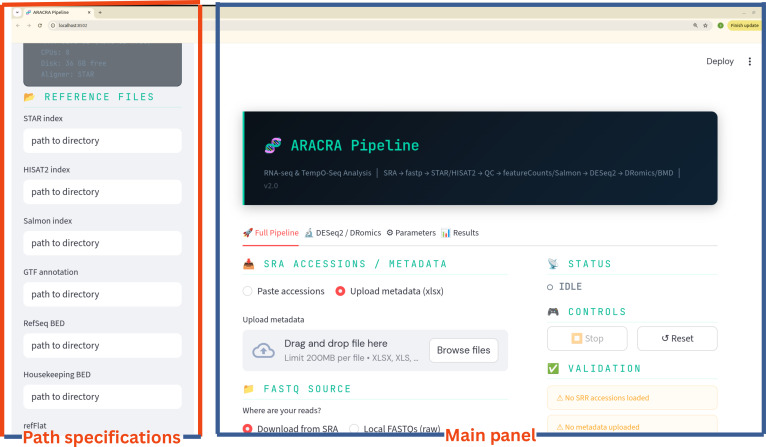
User interface of ARACRA. The interface includes a side panel for reference paths and execution settings and a main panel organized into 4 tabs: Full Pipeline (data input and processing), DESeq2/DRomics (statistical and dose–response analysis), Parameters (customization), and Results (visualization and downloadable outputs).

A Streamlit-based web interface is provided for interactive usage and visualization (Fig. 3). The interface is organized into 2 sections, i.e., the side panel provides reference file paths and execution profiles installed during the setup phase, and a main panel, designed to handle core pipeline with suite of tools and is further divided into 4 tabs.

Full Pipeline Tab: This tab allows the users to retrieve data from SRA or even local file directory through accession numbers provided in the metadata and run it through the entire pipeline—Quality Control, Alignment, and Quantification to produce raw gene counts. For each step, it allows the user to configure different tools available in the pipeline.

DESeq2/DRomics tab: Raw gene counts from previous tabs are transferred here for statistical analysis of identifying DEGs and Dose–Response modeling. Users can also upload previously analyzed count data as well, along with the metadata.

Parameters: This tab allows users to fine-tune and customize parameters being used in statistical analysis. During setup, it is prefilled with default values as suggested in Supplementary Materials File S2 but can be modified as per the requirements.

Results: Once a run completes, the Results tab shows the analysis results including plots such as PCA, volcano, and list of DEGs, which can be downloaded directly from the tab. For DRomics as well, all the results are displayed including dose–response curves, tPODs calculated using different approaches, and their comparison. For both sections, quality metrics and an overall multiQC report are also available for download and further inspection.

## Result and Discussion

ARACRA was developed to address some of the key limitations in current RNA-seq toxicogenomic workflows by providing standardized, end-to-end analysis. Developed within the EU-PARC initiative (European Partnership for the Assessment of Risks from Chemicals), a pan-European partnership launched in 2022 to advance next-generation, mechanistically grounded chemical risk assessment and reduce reliance on animal testing [41]. Within PARC, Work Package 5 focuses on developing NAMs that fill data gaps in AOPs and enable grouping, read-across, and integrated approaches to testing and assessment for regulatory use [42]. One of the barriers to the regulatory uptake of transcriptomics within this effort is the lack of harmonized, transparent, and reproducible analytical workflows. By promoting transparent processing, metadata standardization, and cross-study comparability, ARACRA ensures traceability, reproducibility, and interpretability across all analytical steps for both bioinformaticians and noncoding domain experts.

All the tools incorporated in ARACRA were carefully selected through a pragmatic, workflow-oriented process, relatability to the domain, and ease of access (Supplementary Materials File S1.1). It does not introduce a new statistical method; rather, its contribution lies in the structured orchestration of established tools coupled to an interactive review layer that preserves expert oversight at critical decision points. Considering that one of the major issues in RNA-seq data analysis is reproducibility and false positives [43], every parameter used, exclusion and inclusion decision of the samples, and intermediate result is preserved in structured JSON outputs for reproducibility and efficient preprocessing protocol established on the foundations of regulatory pipelines with user review. To accomodate user’s provided counts, ARACRA currently provides direct analysis tab where the count matrix is directly analyzed for statistical inference after being harmonised for gene symbols through mapping to official gene symbols and configurable parameters threshold with human oversight. As the complete workflow is open-source, users can also modify the main structure as per the expertise or adapt the data as per current implementation.

To evaluate its end-to-end functionality and computational feasibility, ARACRA was applied to a publicly available HTTr dataset from Beal *et al*. [22], comprising 286 TempO-Seq samples from MCF-7 cells exposed to BPA and 11 data-poor alternatives (GEO: GSE271332). Raw reads were retrieved directly from SRA, processed through the full pipeline, and a quantified gene-expression matrix was generated for downstream DEG analysis and BMD modeling. Phase 1 was completed within 4 h on a workstation with 8 cores and 64-GB RAM; Phase 2 for each chemical–vehicle control pair was completed within 2 h. The vehicle control used in this study was dimethyl sulfoxide or MeOH, depending on the chemical. Quality Control report was generated using multiQC which is available as supplementary file S3_multiqc.html. Detailed description can be found in Supplementary Materials File S1.3.

For BPA, ARACRA identified 1,181 dose-responsive genes from 11,889 analyzed (9.9%), of which 282 BMDs were retained after applying NTP max-dose and extrapolation filters alongside the European Food Safety Authority BMDU/BMDL ratio filter (threshold < 40). The BMDU/BMDL filter was the dominant quality criterion, removing 62.5% of fitted BMDs from the analysis and consistent with bootstrap CI imprecision typical at omics scale where uncertain and wide bootstrap CIs (large BMDU/BMDL) in the BMDs of the gene affect the overall tPOD of the chemical [34]. For 2,4′-BPA, 404 quality-filtered BMDs were retained from 1,459 dose-responsive genes, reflecting greater transcriptomic activity than BPA, corroborated by a lower Rank25 tPoD (0.0185 μM *vs.* 0.0361 μM; [40]). Across all 12 chemicals, the potency ranking was concordant with Beal *et al*. [22], with ARACRA’s Rank25 estimates falling within the reported BMCL–BMCU CIs for 8 of 10 chemicals with derivable tPoDs (Table 1). The primary discrepancy was observed for resorcinol sulfide, for which a Rank25 tPoD could not be derived due to fewer than 25 genes passing quality filters reflecting the combined stringency of the BMDU/BMDL and max-dose filters in ARACRA rather than a biological difference, as Beal *et al*. used BMD Express 3.2 for their BMC calculations, which uses its own model-fitting and filtering framework and resorcinol sulfide was also found to be lowest in potency by them.

**Table 1.** Range of tPoDs derived from ARACRA for BPA and alternatives. The table lists number of dose-responsive genes, successfully fitted models, and quality-filtered BMD estimates for each compound. Reported tPoD ranges and 25th-ranked gene BMD values indicate relative transcriptional potency, with lower values reflecting higher biological activity. The tPOD range represents lower and upper limit of BMD determined as per different methods.

Chemicals	Genes selected	Models fitted	Quality filters passed	tPOD range (μM)	25th ranked gene BMD (μM)
2,4′-Bisphenol A	1,459	1,127	404	0.0151–0.563	0.0185
Bisphenol A	1,181	892	282	0.0111–0.689	0.0361
Bisphenol G	457	341	319	0.1437–0.627	0.209
Rosalic acid	607	506	461	0.2958–4.067	0.296
Bisphenol P	150	137	111	0.1773–0.614	0.253
Tetra methyl bisphenol S	185	166	156	0.7561–9.478	2.104
1,1,2,2-Tetrakis(p-hydroxyphenyl)ethane	195	146	136	0.7878–7.255	1.611
Phenol, 2,2′-ethylidenebis[4,6-bis(1,1-dimethylethyl)-]	36	33	33	1.3629–10.156	10.156
Resorcinol sulfide	18	14	13	1.5678–8.842	N/A
Bisphenol BP	8	3	1	N/A	N/A
Tetramethylol bisphenol A	0	0	0	N/A	N/A

tPODS, transcriptomic points of departure; BPA, bisphenol A; BMD, benchmark dose; N/A, not applicable

Among the 50 estrogen receptor alpha (ERα)-associated genes in the Corton *et al*. biomarker signature [44], 38 were identified as concentration-responsive to BPA and 36 to 2,4′-BPA. The 2 analogues showed high concordance, with 34 biomarker genes common to both and only 2 responsive uniquely to 2,4′-BPA (LIMA1, RAB27B), This overlap with an ER biomarker indicates that both bisphenol analogs engage a common ERα-mediated transcriptional program in MCF-7 cells. For a subset of these genes, including several ERα targets, the estimated BMDs fell below the lowest tested concentration, indicating extrapolation beyond the experimental dose range and may require further validation. File S7.csv lists the genes involved in both chemicals.

The biological relevance of these outputs was further supported by the identity of DEGs. Among the most consistently DEGs identified across transcriptionally active chemicals were growth regulating estrogen receptor binding 1 (GREB1) and progesterone receptor (PGR). GREB1 acts as a coactivator for ERα, stabilizing interactions between ER and other cofactors, and modulating the expression of ER target genes, and therefore, alteration in expression of GREB1 can be a potential cause of endocrine disruption resulting in increased cell proliferation [45]. PGR gene encodes for a nuclear receptor for progesterone, a key steroid hormone involved in reproductive tissue differentiation, ovulation, and pregnancy. PGR was also identified among the top DEGs in the original study by Beal *et al*. [22] at 1 μM and above. Both GREB1 and PGR are induced by estrogen via ERα, and their disruption by EDCs can lead to impaired cell cycle regulation and other processes [46]. However, to validate these results and identify BMD for ERα genes activated at lower doses, further investigations are required. Complete list of DRomics results can be found in the supplementary folder (BMDs). To confirm applicability beyond targeted assays, ARACRA was additionally validated on a whole RNA-seq dataset of BPA-exposed hESCs (Ni *et al*. [47]; Supplementary Materials File S1), where the analysis yielded 335 DEGs enriched for developmental and neurotoxicity-related pathways (Supplementary Materials File S1.3).

The tPODs derived from ARACRA are intended to support chemical risk assessment as the first component of broader assessment chain. A tPOD represents the lowest concentration at which a concerted change in gene expression is detectable and because molecular perturbations precede apical toxicity, tPODs have been proposed as conservative, mechanism-agnostic surrogates for the apical effect levels traditionally used in risk assessment [48]. Within this paradigm, an *in vitro* tPOD (in μM) is not itself a health-based reference value; rather, it is combined with high-throughput toxicokinetic modeling to derive an administered equivalent dose (mg/kg-bw/day) using *in vitro*-to-*in vivo* toxicokinetic extrapolation. ARACRA generates the transcriptomic component of this chain, while the downstream toxicokinetic extrapolation and exposure comparison remain the responsibility of the risk assessor and are not performed within the pipeline. Thomas *et al*. [49] in their study found that short-term exposure of chemicals and tPODs derived can be used for chemical risk assessment as the tPOD derived was substantially correlated with apical POD [49]. EPA Transcriptomics Assessment Products from US EPA (United States Environmental Protection Agency) estimates the use of tPODs and its effective utilization in chemical risk assessment [50].

However, in its current implementation, ARACRA is limited to human reference genome support, restricting direct applicability to rodent toxicogenomic datasets commonly used in regulatory studies. The gene-to-pathway aggregation differs from approaches that model dose–response directly at the gene-set level, such as DoseRider [21], which fits pathway-level mixed-effect models and can thereby capture set-level responsiveness and efficacy rather than inferring it from constituent genes. Also, unlike BMDx2, ARACRA does not currently integrate AOP mapping. Future development will address these gaps through multispecies support, AOP framework integration, and additional statistical modules including edgeR, BMDExpress, weighted gene co-expression network analysis, and gene set enrichment analysis. As the next step, individual steps of the pipeline will be containerized as MCP (Model Context Protocol) server, which will be available for developing AI agent (large language model-based)-assisted transcriptomics analysis where large language models can trigger automated analysis steps using harmonized metadata (using OECD Omics Reporting Framework). This development would let the users through a guided analysis where various datasets which differ in structure and might require expert’s oversight are also analyzed. To evaluate the scalability, the pipeline will also be tested on a wider group of chemicals across different cell lines and species to see how this can be refined over period of time through community engagement.

In context of predictive modeling, uncertainty and sensitivity analysis is one of the essential concepts for reliability and transparency. Though ARACRA does not address uncertainty and sensitivity in an automated manner, it aims for best practices for minimum false positives at 3 stages in Phase 2: selection of genes; model fitting, and BMD estimation, each of which is handled within the DRomics framework. Genes are screened for dose-responsiveness using 1 of the 3 trend tests available in DRomics: a quadratic, linear, or analysis of variance test as per the experimental setup and hypothesis. All 3 methods are fitted internally via an empirical Bayesian approach using DESeq2 for RNA-seq data, with Benjamini–Hochberg false discovery rate correction applied across all tested genes to control the rate of false positives at the selection stage.

At the model fitting stage, 5 dose–response models (linear, exponential, Hill, Gauss-probit, and log-Gauss-probit) are simultaneously fitted to each responsive gene by nonlinear regression using the nls() function. Model selection is performed using the second-order AICc, which applies a sample-size correction to penalize over-parameterized models. Users may alternatively select BIC (Bayesian information criterion) or AIC (Akaike information criterion) as the model criterion via the Parameters tab, though AICc is strongly recommended to prevent overfitting. The sensitivity of gene-level model assignment to this criterion choice can be assessed by comparing outputs generated under different settings, with all parameter choices preserved in the JSON log. A gene is retained as successfully fitted only if the best-fit model improves upon the null (constant) model by a minimum AICc difference of 2 (deltaAICminfromnullmodel), functioning as a sensitivity threshold below which no structured dose–response signal is considered reliably distinguishable from noise.

Parametric uncertainty in BMD estimation is addressed in bmdboot() function from DRomics, generating per-gene CIs on both BMD-zSD and BMD-xfold estimates. The number of bootstrap iterations (default: 1,000) is user-configurable, and bootstrapped CIs are computed as percentiles of the resampled BMD distribution (2.5th to 97.5th percentiles for 95% CIs). When bootstrap CIs are computed, ARACRA applies a cascade of postfitting quality filters recommended by the NTP (2018) framework and consistent with European Food Safety Authority guidance on BMD analysis: (a) exclusion of BMDs exceeding the maximum tested dose (extrapolation filter); (b) exclusion of BMDs falling below the lowest nonzero tested dose divided by the extrapolation factor (default: 10); and (c) exclusion of BMDs where the BMDU/BMDL ratio exceeds a user-defined threshold (default: 40), directly filtering out genes whose BMD uncertainty is too large to support reliable tPOD contribution.

However, ARACRA does not quantify additional uncertainty and might require user’s oversight. These include choice of alignment tool (STAR or HISAT2), quantification method (featureCounts or Salmon), and normalization strategy that might affect gene-count matrix owing to changes in algorithms. Also, the pathway-level tPOD is sensitive to the gene-set annotation database used (GO, KEGG, MSigDB Hallmark, and MSigDB C2), and tPODs for the same chemical may differ across databases depending on pathway gene membership; users should compare tPODs across annotation sources rather than relying on a single database.

## Conclusion

ARACRA provides an easy-to-use platform that can be configured locally for end-to-end RNA seq analysis and used in the field of chemical risk assessment. It eliminates the need for manual command line actions with an interactive user interface for overall review by the experts, thereby making RNA-seq analysis easier for noncoding researchers. It also provides reproducibility and transparency by storing the results and parameters used for future reference and downstream reporting.

## Data Availability

High-throughput RNA-seq data was used from GEO database under the accession number GSE271332. All the codes used in this work is available on GitHub: InSilicoVida-Research-Lab/ARACRA along with a user manual for step-by-step installation and description. Researchers can use the demo data (S4_metadata.csv) or even upload their own data to do the analysis.
