# Supplementary material for: ARACRA: Automated RNA-seq Analysis for Chemical Risk Assessment
Source: Comput Struct Biotechnol J. 2026 Aug 19;35(1):0186. doi: 10.34133/csbj.0186 (PMC13486726; doi:10.34133/csbj.0186)
Supplement: Supplementary file 1 [file csbj.0186.f1.zip › S1_cleaned.docx]

# **ARACRA: Automated RNA-seq Analysis for Chemical Risk Assessment**

Shubh Sharma^1^, Saurav Kumar^1,2^, Judit Biosca-Brull^3,4^, Deepika Deepika^1,2^, Vikas Kumar^1,2^*

*^1^* Institut de Recerca Biomèdica Catalunya Sud (IRBCatSud), *Department of chemical engineering, Universitat Rovira I Virgili, Tarragona, Spain*

*^2^German Federal Institute for Risk Assessment (BfR), Berlin, Germany*

^3^Universitat Rovira i Virgili, Pere Virgili Research Institute, Center of Environmental, Food and Toxicological Technology (TECNATOX), Reus, Spain

^4^Universitat Rovira i Virgili, Department of Biochemistry and Biotechnology, Tarragona, Spain

*To whom correspondence should be addressed. Email: [vikas.kumar@urv.cat](mailto:vikas.kumar@urv.cat)

**Supplementary document**

**S1.1 Tools selection:**

For each step in ARACRA, tools were selected as per computational efficiency, reproducibility and ease of integration into workflow. For each stage of analysis, we prioritized established and actively maintained tools that are widely used in the field, generate standardized outputs, and support downstream statistical interpretation. This approach was chosen to minimize technical variability, improve transparency, and ensure that the workflow remains accessible to users with limited programming experience.

Preprocessing

SRA toolkit [1] provides standardized retrieval of public sequencing data from repositories such as GEO and SRA. Fastp [2] is widely used in transcriptomics data analysis for adapter trimming, low-quality base removal, and read filtering in one efficient step, which improves downstream alignment and reduces technical noise, whereas FastQ Screen [3] helps in contamination detection and off-target read mapping against multiple reference genomes to reduce false positives.

Alignment:

STAR [4] and HISAT2 [5] were selected for alignment to reference genome as both are splice-aware aligners. Two aligners are provided for optimal use of computational resources where HISAT2 can be configured with system with low resources (<32 GB RAM) while STAR requires high computational resources (>32 GB RAM).

Quantification:

For quantification, featureCounts [6] is available for generating gene-level count matrix from aligned reads which is directly compatible with downstream differential expression analysis. Also, Salmon [7], which provides fast and bias-aware expression quantification is available to enhance and speed up the analysis.

Post Alignment Quality Control:

For post alignment quality control, RSeQC [8], Picard and Qualimap [9] are integrated. RSeQC is used for inferring strandedness, GC bias, read distribution and transcripts integrity in RNA-seq data. Whereas, Picard summarizes alignment quality and calculated rate of duplicate reads. Qualimap can be used to estimate genomic coverage profiles, mapping qualities.

Statistical Processing:

For statistical analysis, ARACRA integrates DESeq2 [10] as the main package for identifying differentially expressed genes. Whereas, for dose response modelling, the pipeline integrates DRomics [11], designed specifically for omics-based dose-response analysis and benchmark dose estimation along with tcplfit [12].

ARACRA is based on similar framework-level logic as R-ODAF [13] and does share some of the same tools but adapts the final software stacks to its own end-to-end scope and data types to increase transparency and reproducibility.

ARACRA ensures best practices to be followed while performing the analysis at different stages of pipeline [14]. It maintains consistent flow of data in compatible format across all tools and ensures correct mapping through the gene symbols. For reproducibility and traceability, it also produces JSON files of the parameters used across all tools and maintains logs. While passing the data for statistical analysis through DESeq2 and DRomics it ensures that over correction does not take place through ComBat-Seq [15] and that raw counts (after filtering and outlier removal) is passed and DESeq2 uses batch as a covariate along with treatment for correct results. For Dose-response modelling, it guides the users for different type of tPOD estimation available and filters applied through different criterias.

| **Tool selected** | **Rationale for selection** | **Alternatives considered** | **Why not selected** |
| --- | --- | --- | --- |
| fastp (1.3.0) | Single-pass, multithreaded C++; combines adapter detection, quality trimming, filtering and QC reporting in one tool; JSON output integrates directly with MultiQC | Trimmomatic; Cutadapt; Trim Galore | Trimmomatic is Java-based and slower, needs explicit adapter files and a separate QC step; Cutadapt/Trim Galore require pairing with a separate QC tool, whereas fastp is all-in-one |
| FastQ Screen | Detects cross-species/rRNA/adapter contamination against multiple reference genomes before alignment | manual BLAST | Breaks the workflow and automation |
| STAR (2.7.11b)/ HISAT2 | HISAT2Splice-aware alignment producing BAMs required for coverage and contamination QC; STAR for maximum sensitivity (>90% unique), HISAT2 as a lower-memory splice-aware option | Bowtie2 | Not splice aware |
| Salmon / featureCounts | featureCounts and Salmon were selected to cover two complementary quantification routes within the same workflow. featureCounts provides fast, alignment-based gene-level counting directly from the STAR/HISAT2 BAM outputs, yielding integer counts that are natively compatible with DESeq2. Salmon provides an alignment-free route, generating bias-corrected transcript-level estimates directly from reads, imported via tximport for gene-level differential expression. | RSEM; HTSeq-count | Offering both allows the pipeline to serve alignment-based and alignment-free analyses within a single, consistent DESeq2 framework. |
| ComBat-seq (sva 3.54.0) | Negative-binomial model designed for RNA-seq counts; returns integer counts, preserving compatibility with DESeq2 downstream | ComBat (original); limma removeBatchEffect | Original ComBat assumes continuous/log data; limma removeBatchEffect outputs a modified expression matrix (not counts), breaking DESeq2 input; |
| DESeq2 (1.46.0) | Robust with small replicate numbers typical of toxicogenomic designs; shrinkage-based dispersion and LFC estimation; widely validated and cited | edgeR; limma-voom | edgeR is comparable but DESeq2's default normalisation/shrinkage was preferred; limma-voom is better suited to large sample sizes than the modest n per dose here |
| DRomics (2.6.2)/  tcplfit2 | R-native, purpose-built for omics dose-response; fits and selects among multiple monotonic and biphasic models; integrates into the R workflow and supports distribution-based tPODs | BMDExpress 2; PROAST; bmdrc | BMDExpress 2 is a Java GUI, hard to embed in a Nextflow/R pipeline; PROAST is oriented to apical BMD and GUI-driven; bmdrc is narrower in model scope |
|  |  |  |  |

| **Abbreviation** | **Full Form** |
| --- | --- |
| **Workflow & assay** |  |
| ARACRA | Automated RNA-seq Analysis for Chemical Risk Assessment |
| RNA-seq | RNA sequencing |
| HTTr | High-Throughput Transcriptomics |
| TempO-Seq | Templated Oligo assay with Sequencing readout |
| NAM | New Approach Methodology |
| QC | Quality Control |
| DEG | Differentially Expressed Gene |
| DR | Dose–Response |
| RA | Risk Assessment |
| **Dose–response & statistics** |  |
| PoD (tPoD) | Point of Departure (transcriptomic Point of Departure) |
| BMD | Benchmark Dose |
| BMDL / BMDU | Benchmark Dose Lower / Upper confidence limit |
| BMC | Benchmark Concentration |
| BMCL / BMCU | Benchmark Concentration Lower / Upper confidence limit |
| BMR | Benchmark Response |
| AICc | Akaike Information Criterion, corrected for small sample size |
| FDR | False Discovery Rate |
| log2FC | log2 Fold Change |
| CPM | Counts Per Million |
| VST | Variance Stabilising Transformation |
| PCA | Principal Component Analysis |
| CI | Confidence Interval |
| SD | Standard Deviation |
| IQR | Interquartile Range |
| **Pathway & gene-set resources** |  |
| GO | Gene Ontology |
| KEGG | Kyoto Encyclopedia of Genes and Genomes |
| MSigDB | Molecular Signatures Database |
| GSEA | Gene Set Enrichment Analysis |
| WGCNA | Weighted Gene Co-expression Network Analysis |
| AOP | Adverse Outcome Pathway |
| **Data repositories & formats** |  |
| SRA | Sequence Read Archive |
| GEO | Gene Expression Omnibus |
| **Reference genome** |  |
| GRCh38 / hg38 | Genome Reference Consortium Human build 38 |
| GENCODE | Reference gene annotation set for human/mouse genomes |
| **Frameworks, organizations & standards** |  |
| R-ODAF | Regulatory Omics Data Analysis Framework |
| EU-PARC | European Partnership for the Assessment of Risks from Chemicals |
| EFSA | European Food Safety Authority |
| NTP | National Toxicology Program |
| EPA | (U.S.) Environmental Protection Agency |
| OECD | Organisation for Economic Co-operation and Development |
| DSL2 | Domain-Specific Language 2 (Nextflow) |
| GUI | Graphical User Interface |
| MCP | Model Context Protocol |
| LLM | Large Language Model |

Table S1: Acronyms and full form used

| Name | **Tool** | **Version** |
| --- | --- | --- |
| **Framework & interface** | Nextflow (DSL2) | 25.10.4 |
|  | R | 4.4.3 |
|  | Python | 3.12.13 |
|  | Streamlit | 1.55.0 |
| **Data retrieval** | SRA Toolkit | 3.2.1 |
| **Pre-alignment QC** | fastp | 1.3.0 |
|  | FastQ Screen | 0.16.0 |
| **Alignment** | STAR | 2.7.11b |
|  | HISAT2 | 2.2.2 |
| **Quantification** | Subread (featureCounts) | 2.1.1 |
|  | Salmon | 1.11.4 |
| **Post-alignment QC** | RSeQC | 5.0.4 |
|  | Picard | 3.4.0 |
|  | QualiMap | 2.3 |
|  | SAMtools | 1.23.1 |
| **Reporting** | MultiQC | 1.33 |

Table S2: Different tools used along with the versions

| **Package** | **Version** |
| --- | --- |
| DESeq2 | 1.46.0 |
| DRomics | 2.6.2 |
| sva (ComBat-Seq) | 3.54.0 |
| edgeR | 4.4.0 |
| limma | 3.62.1 |
| org.Hs.eg.db | 3.20.0 |
| Rsamtools | 2.22.0 |
| ggplot2 | 4.0.2 |

**S1.2 Reference index construction:**

ARACRA builds reference indices once during the setup phase (setup.sh) and reuses them across runs. Two index types are supported depending on the assay: whole-transcriptome RNA-seq uses a genome/transcriptome index, whereas targeted TempO-Seq uses a probe-level reference built dynamically from the assay manifest. In the current version, human references are based on the GRCh38 (hg38) primary assembly with GENCODE v44 annotation; multi-species support is planned for the next release.

For RNA-seq, three indices are prepared. A splice-aware STAR index is generated from the genome FASTA and the GENCODE GTF (STAR --runMode genomeGenerate … --sjdbOverhang 100), built only on systems with ≥ 32 GB RAM; on lower-memory systems this step is skipped and HISAT2 is used instead . For HISAT2, the pre-built, transcriptome-aware GRCh38 genome_tran index distributed by the HISAT2 authors is downloaded directly, with splice sites and exons already incorporated . For Salmon, a decoy-aware index is constructed from a "gentrome" — the GENCODE transcriptome concatenated with the genome sequence as decoy — which improves quantification specificity by absorbing reads of genomic origin [16]. Full commands are provided in setup.sh on the GitHub repository.

For targeted TempO-Seq, the pipeline uses a fundamentally different, dynamic indexing strategy, because the assay reads correspond to a restricted set of ~50-nucleotide, target-specific detector-oligonucleotide probes rather than the whole transcriptome. Probe-level mapping and quantification is the native readout for TempO-Seq [17] [18]; aligning these reads to the full genome would be computationally inefficient and could introduce off-target or multi-mapping artefacts. During execution, the pipeline parses the user-provided BioSpyder manifest into a compact FASTA containing only the probe sequences — for the human whole-transcriptome panel used here, 22,533 probes of uniform 50 nt length, covering 19,683 genes, for a total reference length of ≈ 1.13 Mb — and builds a STAR index directly from it. Because STAR's default suffix-array parameter is too large for so small a reference (which STAR reports as a potential cause of segmentation faults at the mapping step), the pipeline automatically scales it down following the STAR manual's small-genome rule, --genomeSAindexNbases = min(14, ⌊log₂(L)/2 − 1⌋), yielding a value of 9 for this panel.

Reads are then mapped to the probe index with STAR parameters tuned for probe-level quantification: spliced alignment is disabled (--alignIntronMax 1), only uniquely-mapping reads are retained (--outFilterMultimapNmax 1), and mismatches are restricted to at most two per read (--outFilterMismatchNmax 2; --outFilterMismatchNoverLmax 0.05). As the reads (51 bp) are one base longer than the probes (50 nt), STAR's default local alignment soft-clips the trailing base, so the single-base overhang is excluded rather than scored as a mismatch. Finally, per-probe read counts are extracted with samtools idxstats and aggregated to gene level — summing all probes mapping to the same gene and resolving identifiers with an ENSEMBL-preferred convention — to produce the gene × sample count matrix used downstream by DESeq2 and DRomics.

Table S3: Different thresholds used at different stages of pipeline along with the default values which can be configured accordingly.

| **Pipeline Step** | **Metric** | **Threshold** | **Tool / location** | **Source** |
| --- | --- | --- | --- | --- |
| Pre-alignment | Qualified base Phred | ≥ Q15 | fastp | fastp default |
| Pre-alignment | Max unqualified bases per read | ≤ 40% | fastp | fastp default |
| Pre-alignment | Min read length after trimming | ≥ 15 bp | fastp | fastp default |
| Pre-alignment | Contamination screen | reported, reviewed at QC gate (not auto-filtered) | FastQ Screen | ARACRA |
| Read quality | % bases ≥ Q30 (after trimming) | ≥ 70% | parse_fastp_qc.py (fastp JSON) | **R-ODAF** (Verheijen 2022) |
| Read quality | Forward–reverse Q30 balance | Δ ≤ 25% (paired-end only; N/A for single-end) | parse_fastp_qc.py | **R-ODAF** (Verheijen 2022) |
| Alignment | Overall aligned rate (RNA-seq) | < 70% flags sample | qc_outlier_check.py (--align_floor 70) | **R-ODAF** (Verheijen 2022) |
| Alignment | Overall aligned rate (TempO-Seq) | floor disabled; assignment rate reviewed | STAR / qc_outlier_check.py | ARACRA |
| Alignment | Probe multimapping (TempO-Seq) | unique only (--outFilterMultimapNmax 1) | STAR | ARACRA |
| Alignment | Mismatches per read (TempO-Seq) | ≤ 2 and ≤ 5% of mapped length | STAR | ARACRA |
| Alignment | Mapping-rate outlier (batch-relative) | Grubbs (α=0.05) **and** IQR (1.5×) both flag; n ≥ 3 — flagged if floor **or** this triggers | qc_outlier_check.py | ARACRA |
| Alignment | Min library size | ≥ 1,000,000 reads | utils.R (read_thresh) | ARACRA |
| Strandedness | Forward/reverse call | sense fraction > 0.75 | RSeQC infer_experiment | ARACRA |
| Post-alignment | Exonic %, rRNA %, genes detected | reported via MultiQC, reviewed (no fixed cutoff) | RSeQC / Picard / Qualimap | ARACRA |
| Expression QC | PCA outlier (per group, ≥ 3 samples) | distance from group centroid > mean + 2·SD on PC1–PC2 (VST) | utils.R detect_pca_outliers | ARACRA |
| Expression QC | Batch correction | ComBat-Seq (group=Type, shrink=FALSE); PCA-viz only — Batch as DESeq2 covariate for DEG | utils.R apply_batch_correction | ARACRA |
| Gene filter | Low-expression CPM relevance | ≥ 1 CPM in ≥ 75% of samples of ≥ 1 group | utils.R filter_samples_and_genes | **R-ODAF** (Verheijen 2022) |
| Gene selection (DR) | Responsive-gene trend test | quadratic, FDR ≤ 0.05 | DRomics itemselect | Larras 2018 |
| DEG significance | Default DEG call | padj < 0.05 and \|log2FC\| > 1.0 | run_deseq2.R | ARACRA |
| DEG significance | R-ODAF mode (optional) | --rodaf_deg: padj < 0.01, no FC filter | run_deseq2.R | **R-ODAF** (Verheijen 2022) |
| Dose–response | BMR | z = 1 (1 SD), x = 10% | run_dromics.R (bmd_z, bmd_x) | NTP 2018 |
| Dose–response | BMD confidence interval | bootstrap, 1000 iterations (on for reported BMDU/BMDL) | DRomics bmdboot | Larras 2018 |
| Dose–response | BMD quality filters | BMDU/BMDL < 40; max-dose; extrapolation ×10; fold-change min (default 0) | run_dromics.R | EFSA / NTP 2018 |

**S1.3 Validation:**

The dataset having accession number GSE271332 was analyzed using ARACRA using the metadata information S4_metadata.csv. The dataset had Tempo-Seq HTTr data of BPA and 11 alternatives tested at different concentrations in 3 batches as per Beal *et* al., 2024 [19]. In total, 286 samples were present, raw data of which was retrieved from SRA database. According to Beal *et* al., 2024, MCF-7 cells were cultured in phenol red-free medium with charcoal-dextran-stripped FBS and exposed for 48 h, in triplicate wells, to BPA and eleven structurally related data-poor alternatives at five nominal concentrations (0.5, 1, 5, 10, 25 µM), alongside vehicle controls (1% DMSO or 1% MeOH; BPBP and THPE were dissolved in MeOH, the remainder in DMSO). Following exposure, cells were washed with PBS and lysed in 2× TempO-Seq Enhanced Lysis Buffer. Gene expression was measured with the TempO-Seq Human Whole Transcriptome Panel v2.1 (Index E, F, and G, corresponding to the three sequencing batches) and sequenced on an Illumina NextSeq 2000 (2×50 bp P3 flow cell, single-end), yielding a median depth of ~3.3 million uniquely mapped reads per sample. The manifest file was used to build the local reference index against with the reads were aligned with STAR.

**S1.3.1 Overall QC report from MultiQC revealed following information:**

Fastp filtering retained an average of 99.83% of reads per sample across 286 samples, with negligible adapter contamination (maximum 2.27%) and minimal low-quality or too-short read fractions (<0.15% and <1.3%, respectively), confirming high raw sequencing quality. STAR alignment yielded a mean unique mapping rate of 94.6%, with only 6 samples having unique mapping rate less than 70%. These 6 samples were removed from the further analysis. Library sequencing depth ranged from approximately 0.4 to 7.3 million reads per sample (mean 3.6 million; median 3.7 million), with the majority of libraries (250/286) exceeding 2 million reads. FastQ Screen confirmed predominantly human origin of the sequenced reads, with no detectable contamination from common laboratory organisms. Being TempoSeq data, duplication rate was high as it is probe targeted mechanism of sequencing. A detailed Multi-QC report is attached as S3_multiqc.html.

**S1.3.2 Further Downstream processing:**

Gene-counts matrix was then used for differential gene expression analysis and dose-response modelling. An additional layer quality control step was performed which help the users to filter noise genes in the dataset. As soon as the user select chemical and its corresponding control in the menu, subset of count tables is created specific to samples for those chemicals by mapping through accession numbers between count matrix and metadata. Batch correction was performed using ComBat Seq method and samples were visualized using PCA analysis. Genes having less than 1 CPM within its treatment group were removed from analysis. Approximately 11500 genes passed the threshold per chemical and were considered for DEG and dose response analysis. Subsequent data were retrieved from raw count matrix and utilized by DeSeq2 package with treatment as design (Chemical v/s control) and batch as covariate for identifying differentially expressed genes. Overall results of DEGs identified are summarized in table 1 below:

Table S44: Summary of DEGs when compared overall exposure of chemicals against corresponding control samples.

| **Chemical Name** | **PCA Outliers Removed** | **Total Genes in Matrix** | **Genes After Low-Count Filtering** | **Upregulated** | **Downregulated** | **Dose Levels Tested** |
| --- | --- | --- | --- | --- | --- | --- |
| 1,1,2,2-Tetrakis(p-hydroxyphenyl)ethane | 1 | 19686 | 12017 | 9 | 2 | 5 |
| 2,2'-Methylenebis(4-methyl-6-tert-butylphenol) | N/A | 19686 | 11370 | 346 | 163 | 5 |
| 2,4'-Bisphenol A | 2 | 19686 | 11884 | **554** | 203 | 5 |
| Bisphenol A | 0 | 19686 | 11886 | 497 | 148 | 5 |
| Bisphenol BP | 0 | 19686 | 12063 | 0 | 0 | 5 |
| Bisphenol G | 1 | 19686 | 11582 | 245 | 42 | 5 |
| Bisphenol P | 0 | 19686 | 11498 | 63 | 15 | 5 |
| Phenol, 2,2'-ethylidenebis[4,6-bis(1,1-dimethylethyl)- | 2 | 19686 | 11888 | 0 | 0 | 5 |
| Resorcinol Sulfide | 1 | 19686 | 11690 | 2 | 0 | 5 |
| Rosalic Acid | 0 | 19686 | 11683 | 87 | 8 | 5 |
| Tetra Methyl Bisphenol S | 1 | 19686 | 9607 | 19 | 2 | 5 |
| Tetramethylol Bisphenol A | 0 | 19686 | 7493 | 3 | 6 | 5 |

**S1.3.3 Case study: Exposure of BPA to hESCs**

Along with Tempo-Seq analysis, the pipeline was also tested on whole RNA-seq dataset of BPA-exposed hESCs from Ni *et* al., 2024 [20]. Metadata was created with user specified ID along with dosing and batch information along with sequencing layout. In this case, the sequencing was in paired end layout and further alignment was done using paired end processing option. For all the samples, adaptor contamination was minimal, and alignment rate was greater than 70%. Gene expression was quantified and used for DEG analysis.

Analysis with DESEQ2 revealed 335 genes to be differentially expressed having adjusted p-value less than 0.05 and log2FC less than –1 and greater than +1. Out of these, 43 genes were downregulated (DKK4, SMIM43, PROK2, OLR1, CYP1A1, SOX1 and more) and 292 were upregulated (FMOD, RGS13, HOXB5, DNAH2, HGF, CCN4 and many more). Functional annotation and GO enrichment of these genes using gProfiler was done to identify altered biological pathways and processes. Fig S1 shows major pathways and biological processes involved. GO terms enrichment reveals major biological processes involved are related to tissue development, nervous system development and many cellular processes (Fig. S1a). While KEGG enrichment shows pathways like ovarian steroidogenesis, Extracellular matrix (ECM)-receptor interaction, phosphatidylinositol 3-kinase (PI3K)-AKT signaling pathway, and many more (Fig. S1b). Earlier studies also report BPA involvement in embryonal neurotoxicity, impaired nervous-system development and brain cytoarchitecture and potential effects on Blood-Brain-Barrier integrity [21] [22]. Also, early postnatal BPA exposure alters axon growth and social locomotor behaviour and zebrafish studies also reported adverse neurodevelopmental effects and disrupted craniofacial cartilage development [23] [24]. However, full mechanistic characterization of BPA’s mode of action will require further investigation.

Fig S1 Pathway enrichment and functional annotation of differentially expressed genes identified from the analysis using G:profiler API. A) Biological processes significantly affected on exposure of BPA to hESCs, enriched by GO having p-value less than 0.05. B) Biological pathways significantly affected on exposure of BPA to hESCs, enriched by KEGG database having p-value less than 0.05.

Table S7: List of samples excluded from the analysis as per PCA visualisation and flagged outliers

| **Chemical** | **Condition** | **Accession** |
| --- | --- | --- |
| Bisphenol A | Treated 0.5µM | SRR29684829 |
| Bisphenol P | Treated 10µM | SRR29684981 |
| Bisphenol G | Treated 5µM | SRR29684833 |
| THPE | Treated 5µM | SRR29684976 |
| Resorcinol sulfide | Treated 0.5µM | SRR29684855 |
| Rosalic Acid | Treated 0.5µM | SRR29684916 |
| Tetramethylol Bisphenol A | Treated 0.5µM | SRR29685101 |
| Tetra Methyl Bisphenol S | Treated 5µM | SRR29685106 |
| Isonox129 | Treated 5µM | SRR29685028 |

References:

1. https://github.com/ncbi/sra-tools/wiki
2. 2. Shifu Chen, Yanqing Zhou, Yaru Chen, Jia Gu, fastp: an ultra-fast all-in-one FASTQ preprocessor, Bioinformatics, Volume 34, Issue 17, September 2018, Pages i884–i890, <https://doi.org/10.1093/bioinformatics/bty560>
3. Wingett SW, Andrews S. FastQ Screen: A tool for multi-genome mapping and quality control. F1000Res. 2018 Aug 24;7:1338. doi: 10.12688/f1000research.15931.2. PMID: 30254741; PMCID: PMC6124377.
4. Kim, D., Paggi, J.M., Park, C. et al. Graph-based genome alignment and genotyping with HISAT2 and HISAT-genotype. Nat Biotechnol 37, 907–915 (2019). <https://doi.org/10.1038/s41587-019-0201-4>
5. Dobin A, Davis CA, Schlesinger F, Drenkow J, Zaleski C, Jha S, Batut P, Chaisson M, Gingeras TR. STAR: ultrafast universal RNA-seq aligner. Bioinformatics. 2013 Jan 1;29(1):15-21. doi: 10.1093/bioinformatics/bts635. Epub 2012 Oct 25. PMID: 23104886; PMCID: PMC3530905.
6. Yang Liao, Gordon K. Smyth, Wei Shi, featureCounts: an efficient general purpose program for assigning sequence reads to genomic features, Bioinformatics, Volume 30, Issue 7, April 2014, Pages 923–930, <https://doi.org/10.1093/bioinformatics/btt656>
7. Patro R, Duggal G, Love MI, Irizarry RA, Kingsford C. Salmon provides fast and bias-aware quantification of transcript expression. Nat Methods. 2017 Apr;14(4):417-419. doi: 10.1038/nmeth.4197. Epub 2017 Mar 6. PMID: 28263959; PMCID: PMC5600148.
8. Liguo Wang, Shengqin Wang, Wei Li, RSeQC: quality control of RNA-seq experiments, Bioinformatics, Volume 28, Issue 16, August 2012, Pages 2184–2185, <https://doi.org/10.1093/bioinformatics/bts356>
9. Fernando García-Alcalde, Konstantin Okonechnikov, José Carbonell, Luis M. Cruz, Stefan Götz, Sonia Tarazona, Joaquín Dopazo, Thomas F. Meyer, Ana Conesa, Qualimap: evaluating next-generation sequencing alignment data, Bioinformatics, Volume 28, Issue 20, October 2012, Pages 2678–2679, <https://doi.org/10.1093/bioinformatics/bts503>
10. Love, M. I., Huber, W., & Anders, S. (2014). Moderated estimation of fold change and dispersion for RNA-seq data with DESeq2. Genome biology, 15(12), 550.
11. Larras F, Billoir E, Baillard V, Siberchicot A, Scholz S, Wubet T, Tarkka M, Schmitt-Jansen M, Delignette-Muller ML. DRomics: A Turnkey Tool to Support the Use of the Dose-Response Framework for Omics Data in Ecological Risk Assessment. Environ Sci Technol. 2018 Dec 18;52(24):14461-14468. doi: 10.1021/acs.est.8b04752. Epub 2018 Dec 4. PMID: 30444611.
12. Sheffield T, Brown J, Davidson S, Friedman KP, Judson R. tcplfit2: an R-language general purpose concentration-response modeling package. Bioinformatics. 2022 Jan 27;38(4):1157-1158. doi: 10.1093/bioinformatics/btab779.
13. Verheijen MC, Meier MJ, Asensio JO, Gant TW, Tong W, Yauk CL, Caiment F. R-ODAF: Omics data analysis framework for regulatory application. Regul Toxicol Pharmacol. 2022 Jun;131:105143. doi: 10.1016/j.yrtph.2022.105143. Epub 2022 Mar 3. PMID: 35247516.
14. Conesa, Ana et al. “A survey of best practices for RNA-seq data analysis.” Genome biology vol. 17 13. 26 Jan. 2016, doi:10.1186/s13059-016-0881-8
15. Yuqing Zhang, Giovanni Parmigiani, W Evan Johnson, ComBat-seq: batch effect adjustment for RNA-seq count data, NAR Genomics and Bioinformatics, Volume 2, Issue 3, September 2020, lqaa078, <https://doi.org/10.1093/nargab/lqaa078>
16. Srivastava, Avi et al. “Alignment and mapping methodology influence transcript abundance estimation.” Genome biology vol. 21,1 239. 7 Sep. 2020, doi:10.1186/s13059-020-02151-8
17. Yeakley JM, Shepard PJ, Goyena DE, VanSteenhouse HC, McComb JD, Seligmann BE. A trichostatin A expression signature identified by TempO-Seq targeted whole transcriptome profiling. PLoS One. 2017 May 25;12(5):e0178302. doi: 10.1371/journal.pone.0178302. PMID: 28542535; PMCID: PMC5444820.
18. Harrill, Joshua A et al. “High-Throughput Transcriptomics Platform for Screening Environmental Chemicals.” Toxicological sciences : an official journal of the Society of Toxicology vol. 181,1 (2021): 68-89. doi:10.1093/toxsci/kfab009
19. Beal MA, Coughlan MC, Nunnikhoven A, Gagné M, Barton-Maclaren TS, Bradford LM, Rowan-Carroll A, Williams A, Meier MJ. High-throughput transcriptomics toxicity assessment of eleven data-poor bisphenol A alternatives. Environ Pollut. 2024 Nov 15;361:124827. doi: 10.1016/j.envpol.2024.124827. Epub 2024 Aug 27. PMID: 39197648.
20. Ni, Mengmei et al. “IVIVE-PBPK based new approach methodology for addressing early life toxicity induced by Bisphenol A.” Environmental research vol. 240,Pt 1 (2024): 117343. doi:10.1016/j.envres.2023.117343
21. Rosca, A., Coronel, R., Moreno, M., González, R., Oniga, A., Martín, A., López, V., González, M. D. C., & Liste, I. (2020). Impact of environmental neurotoxic: current methods and usefulness of human stem cells. Heliyon, 6(12), e05773. <https://doi.org/10.1016/j.heliyon.2020.e05773>
22. Haridevamuthu, B., Nayak, S. R. R., Murugan, R., Sudhakaran, G., Pachaiappan, R., Manikandan, K., ... & Arockiaraj, J. (2024). Co-occurrence of azorubine and bisphenol A in beverages increases the risk of developmental toxicity: A study in zebrafish model. Food and Chemical Toxicology, 191, 114861.
23. Huang, W., Wang, X., Zheng, S., Wu, R., Liu, C., & Wu, K. (2021). Effect of bisphenol A on craniofacial cartilage development in zebrafish (Danio rerio) embryos: A morphological study. Ecotoxicol. Environ. Saf, 212(111991), 10-1016.
24. Gyimah, E., Xu, H., Dong, X., Qiu, X., Zhang, Z., Bu, Y., & Akoto, O. (2021). Developmental neurotoxicity of low concentrations of bisphenol A and S exposure in zebrafish. Chemosphere, 262, 128045.
