# Supplementary material for: ARACRA: Automated RNA-seq Analysis for Chemical Risk Assessment
Source: Comput Struct Biotechnol J. 2026 Aug 19;35(1):0186. doi: 10.34133/csbj.0186 (PMC13486726; doi:10.34133/csbj.0186)
Supplement: Supplementary file 1 [file csbj.0186.f1.zip › S6_DR_curves.docx]

# **ARACRA: Automated RNA-seq Analysis for Chemical Risk Assessment**

Shubh Sharma^1^, Saurav Kumar^1,2^, Judit Biosca-Brull^3,4^, Deepika Deepika^1,2^, Vikas Kumar^1,2^*

*^1^IISPV, Hospital Universitari Sant Joan de Reus, Department of chemical engineering, Universitat Rovira I Virgili, Tarragona, Spain*

*^2^German Federal Institute for Risk Assessment (BfR), Berlin, Germany*

^3^Universitat Rovira i Virgili, Pere Virgili Research Institute, Center of Environmental, Food and Toxicological Technology (TECNATOX), Reus, Spain

^4^Universitat Rovira i Virgili, Department of Biochemistry and Biotechnology, Tarragona, Spain

*To whom correspondence should be addressed. Email: [vikas.kumar@urv.cat](mailto:vikas.kumar@urv.cat)

**Supplementary document**

**Dose Response curve generated for top responding genes from chemicals (BPA, BPG, BPP, 2,4’BPA ).**

BPA:

BPG:

BPP

2,4’BPA
